# Sustainable Production of Chitosan from Mussel Shells with Upcycling of Demineralization Effluent into Calcium Formate

**DOI:** 10.3390/ijms27093809

**Published:** 2026-04-24

**Authors:** Chaowared Seangarun, Banjong Boonchom, Somkiat Seesanong, Wimonmat Boonmee, Sirichet Punthipayanon, Nongnuch Laohavisuti, Pesak Rungrojchaipon

**Affiliations:** 1 Material Science for Environmental Sustainability Research Unit, School of Science, King Mongkut’s Institute of Technology Ladkrabang, Bangkok 10520, Thailand; chaowared@gmail.com (C.S.); wimonmat.bo@kmitl.ac.th (W.B.); sirichet@g.swu.ac.th (S.P.); pesak.ru@kmitl.ac.th (P.R.); 2 Department of Chemistry, School of Science, King Mongkut’s Institute of Technology Ladkrabang, Bangkok 10520, Thailand; 3 Municipal Waste and Wastewater Management Learning Center, School of Science, King Mongkut’s Institute of Technology Ladkrabang, Bangkok 10520, Thailand; 4 Office of Administrative Interdisciplinary Program on Agricultural Technology, School of Agricultural Technology, King Mongkut’s Institute of Technology Ladkrabang, Bangkok 10520, Thailand; somkiat.se@kmitl.ac.th; 5 Department of Biology, School of Science, King Mongkut’s Institute of Technology Ladkrabang, Bangkok 10520, Thailand; 6 Department of Sports Science, Faculty of Physical Education, Srinakharinwirot University, Bangkok 10110, Thailand

**Keywords:** chitin, chitosan, calcium formate, mussel shell waste, integrated biorefinery

## Abstract

This study proposes a sustainable, integrated biorefinery approach to valorize mussel shell waste into high-value products, including chitin, chitosan, and calcium formate. Formic acid was employed as an effective demineralizing agent, enabling not only efficient mineral removal but also the direct conversion of the demineralization effluent into value-added calcium formate. The sequential extraction processes, demineralization, deproteinization, and decolorization, successfully yielded purified chitin (PCH), which was subsequently deacetylated to produce chitosan (CTS) with a degree of deacetylation of 85% and a molecular weight of 75 kDa. The physicochemical properties of all products were characterized using Fourier transform infrared spectroscopy (FTIR), X-ray diffraction (XRD), thermogravimetric analysis (TGA), and scanning electron microscopy (SEM). FTIR and XRD analyses confirmed the successful extraction of chitin and chitosan, demonstrating the feasibility of mussel shells as an alternative biopolymer source. In parallel, calcium formate (CCF) was obtained from the demineralization effluent with a yield of 94.19%, and its formation was verified by FTIR and XRD. Elemental analysis by XRF exhibited 98.3% CaO with minimal non-toxic impurities. The TGA/DTG profiles of CCF exhibited a well-defined two-step thermal decomposition, confirming its anhydrous form. Overall, this environmentally benign process enables the simultaneous production of multiple value-added products while significantly improving resource utilization and reducing waste generation. The proposed integrated biorefinery model offers a promising, economically viable pathway for marine biomass valorization, aligned with the Bio-Circular-Green (BCG) economy concept.

## 1. Introduction

The escalating global demand for seafood has led to the accumulation of biogenic waste, such as bivalve shells, constituting a large proportion of environmental problems, with global shell waste generation estimated to exceed 10 million tons per year [1]. In Thailand, the green mussel (*Perna viridis*) is a primary economic bivalve species, and mussel processing generates approximately 30,000 tons of shell waste per year [2]. Conventional disposal methods, such as open-air landfilling or coastal dumping, not only occupy valuable land but also cause leaching of decomposed organic matter, leading to foul odors and promoting the growth of microorganisms [3,4]. Consequently, there is an urgent need to transition from traditional disposal to an integrated biorefinery approach that aligns with the Bio-Circular-Green (BCG) economy model [5]. Mussel shells possess a unique structure in which the mineral phase (approximately 95–97%) is organized into layers of calcite and aragonite, interspersed with a structural scaffolding of chitin (approximately 1–5%) [6,7]. Due to their high calcium carbonate content, mussel shells are widely utilized for the production of various calcium-based materials, including high-purity CaCO_3_ [8], CaO [9], calcium phosphate [10], calcium sulfate [11], calcium acetate [12], calcium lactate [13], and calcium citrate [14]. In contrast, chitin, which constitutes only a minor fraction of the mussel shell, has received considerably less attention [6,7].

Chitin, a biopolymer that consists of β-(1,4)-N acetylglucosamine units, and its primary derivative, chitosan, are recognized for their exceptional biocompatibility, biodegradability, and high nitrogen content [15]. Industrial extraction of chitosan from crustacean-derived raw materials, such as crab and shrimp shells, is typically performed via a series of chemical treatments. These steps usually involve acid demineralization to remove mineral components, basic solution treatment to remove protein, bleaching to eliminate pigments, and, in the deacetylation step, a subsequent highly concentrated basic solution to transform chitin into chitosan [16]. However, the traditional acid demineralization of shell biomass using strong mineral acids, particularly hydrochloric acid (HCl), has some limitations. The use of hydrochloric acid (HCl) generates large volumes of highly saline, corrosive wastewater that requires costly treatment in the industrial chitosan production process [17,18]. Recent studies have demonstrated that organic acids, such as acetic, lactic, and formic acids, serve as effective and sustainable alternatives for demineralization [19,20]. Furthermore, the dissolved calcium ions in the demineralization effluent can be recovered as organic calcium salts, which hold substantial commercial value compared to the waste calcium chloride (CaCl_2_) produced by the HCl route [21]. Our previous research successfully established the protocols for the production of chitosan and calcium acetate (Ca(CH_3_COO)_2_) from mussel shells, proving the viability of this production strategy [21].

Building on this foundation, this study explores the use of formic acid (HCOOH) as a demineralizing reagent for chitin extraction and for the synthesis of calcium formate (Ca(HCOO)_2_). Formic acid offers distinct advantages compared to other organic acids; it has the lowest molecular weight and the highest acidity (pK_a_ = 3.75) among the carboxylic acid series, facilitating a more rapid stoichiometric reaction with CaCO_3_ [22]. Our previous work focused on calcium acetate, which is primarily used as a food preservative or an environmentally friendly deicer [21]. Calcium formate offers a broader range of industrial applications. For example, in the construction sector, calcium formate serves as an effective accelerator of cement hydration, significantly reducing setting time and enhancing early-age compressive strength of concrete, particularly in cold-weather conditions [23,24,25]. Furthermore, in the global livestock industry, calcium formate is highly regarded as a safe and non-corrosive acidifier in animal feed. Calcium formate provides a dual benefit: delivering essential calcium ions while lowering pH to inhibit the growth of bacteria such as *E. coli* and *Salmonella*, thereby improving nutrient absorption and animal growth rates [26]. Conventionally, industrial calcium formate is synthesized through the high-pressure reaction of carbon monoxide (CO) with calcium hydroxide (Ca(OH)_2_) [27]. These synthetic routes often involve energy-intensive conditions and the use of fossil-derived precursors. In contrast, producing calcium formate from mussel shell waste via formic acid demineralization offers environmental and economic benefits over conventional manufacturing. Moreover, integrating calcium formate production into the chitin/chitosan extraction process contributes to minimizing wastewater generation and has the potential to reduce the associated wastewater treatment requirements.

The objective of this research is to evaluate the efficiency of formic acid on the biorefinery process for producing chitin, chitosan, and calcium formate from mussel shells (*Perna viridis*). We suggest that demineralization with formic acid will yield biopolymers while facilitating the direct crystallization of calcium formate from the resulting effluent after demineralization. The products were characterized using FTIR, XRD, TGA, and SEM to confirm their functional groups, crystallinity, thermal stability, and surface morphology, thereby supporting the potential for the sustainable valorization of marine waste. This Bio-Circular-Green (BCG) economy strategy not only provides an alternative source of biopolymers but also addresses the environmental challenges associated with shell waste disposal, meeting the criteria for a sustainable, economically viable circular biorefinery.

## 2. Results and Discussion

### 2.1. Production Results of Chitin and Chitosan

**Figure 1:**
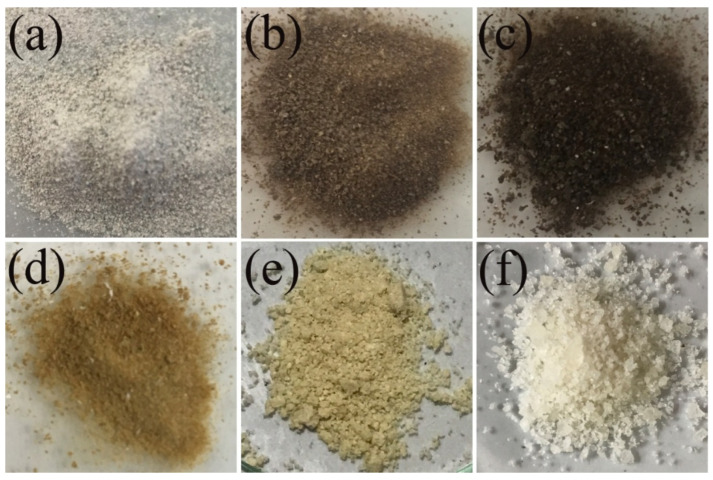
Images of mussel shell powders (MSP) (**a**), raw chitin (RCH) (**b**), deproteinized chitin (DPC) (**c**), purified chitin (PCH) (**d**), chitosan (CTS) (**e**), and calcium formate (CCF) (**f**) derived from mussel shells.

The appearance colors of MSP (a), RCH (b), DPC (c), PCH (d), CTS (e), and CCF (f) samples produced from mussel shells are shown in Figure 1. The appearance colors of all samples exhibit pale gray (MSP), light brown (RCH), dark black (DPC), pale brown (PCH), light yellow (CTS), and pearl white (CCF). The reported mass yields were obtained from three independent experiments and are presented as average values (n = 3). From an initial 100 g of MSP, the mass of the extracted samples consistently decreased across the extraction processes, yielding 3.07 ± 0.05 g, 2.83 ± 0.03 g, 2.75 ± 0.03 g, and 2.20 ± 0.08 g for RCH, DPC, PCH, and CTS, respectively. This progressive decrease in mass is due to the systematic elimination of the inorganic matrix, residual proteins, and pigments, and the conversion of chitin into chitosan after demineralization, deproteinization, decolorization, and deacetylation, respectively. Interestingly, the use of formic acid in the demineralization process resulted in a slightly different color profile and slightly higher yields than those obtained with acetic acid, as reported by Seangarun et al. [21]. The chitosan produced in this work exhibited a degree of deacetylation (DD) of 85%, which is close to the 86% for the acetic acid process [21]. Such a high DD value (above 85%) is critical for enhancing the polymer’s solubility and cationic character, making it highly suitable for specialized applications in drug delivery and antimicrobial coatings [28,29]. Furthermore, the molecular weight (M_w_) of the chitosan, estimated using the Mark–Houwink relationship [η] = KMα, where K and α are empirical constants specific to a given polymer–solvent system at a defined temperature, was approximately 75 kDa. This M_w_ is also similar to that obtained from acetic acid (72 kDa) treatments [21], suggesting that formic acid can also be an efficient chitin extraction reagent during the demineralization process. These results highlight the similar influence of the acid type on the yield, degree of deacetylation (DD), and molecular weight (M_w_) of the extracted biopolymers, while the chemical and physical properties of the obtained products were also characterized by FTIR, XRD, TGA, and SEM, and the results will be shown in the following sections.

### 2.2. Production Results of Calcium Formate

The evaporation of 2000 mL of the demineralization liquid fraction yielded 117.56 ± 1.73 g of calcium formate (CCF). The reaction yield was evaluated using Equation (1), with the calcium ion concentration (0.96 M) serving as the limiting factor. The percentage yield was 94.19%, demonstrating the high efficacy of the formic acid digestion process.

Ca^2+^(aq) + 2HCOO^−^(aq) → Ca(HCOO)_2_(s) (1)

Elemental analysis of the CCF identified by the X-ray Fluorescence (XRF) technique revealed that the obtained product is mainly composed of 98.3% CaO. Moreover, 1.7% of minor trace elements were detected, including Na_2_O (0.59%), MgO (0.06%), Al_2_O_3_ (0.02%), SiO_2_ (0.04%), P_2_O_5_ (0.08%), SO_3_ (0.190%), Cl (0.03%), and SrO (0.69%). This Ca content is significantly higher than that reported for calcium acetate produced from demineralization effluent in previous research (97.8% CaO) [21]. The identification of calcium formate, including its crystalline phase and characteristic functional groups, will be further confirmed by XRD and FTIR, as discussed in the following sections. Unlike several previous studies that focus solely on either chitosan or calcium salt production [30,31,32], this study proposes an integrated biorefinery approach for the simultaneous production of chitin, chitosan, and calcium formate from mussel shell waste. To the best of our knowledge, this is the first study to employ formic acid as a key reagent in such an integrated system, allowing the direct formation of calcium formate from shell-derived calcium. This approach not only simplifies the process but also enhances resource utilization from a single waste source. The efficient yield and purity of the CCF obtained in this study suggest its potential for demanding industrial sectors, such as animal feed additives [33] and cement additives [23,24].

### 2.3. Functional Groups Analysis via FTIR

**Figure 2:**
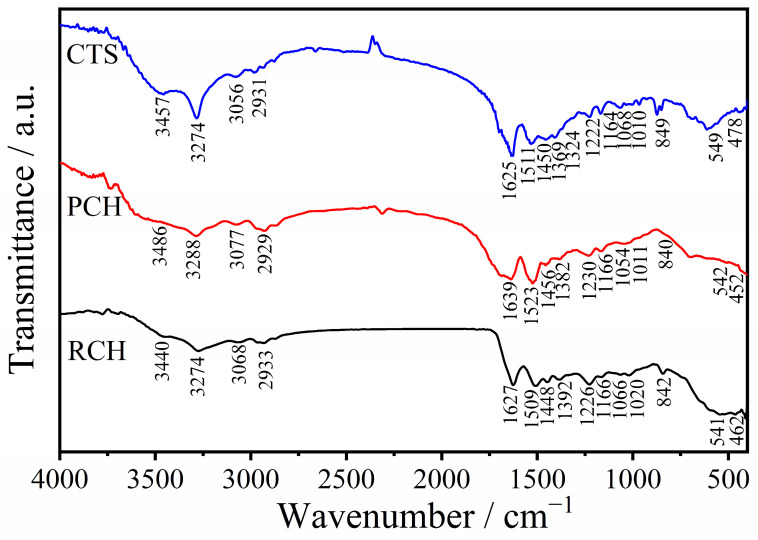
FTIR spectra of raw chitin (RCH), purified chitin (PCH), and chitosan (CTS) samples extracted from mussel shells.

The vibrational characteristics of functional groups of the extracted raw chitin (RCH), purified chitin (PCH), and chitosan (CTS) were analyzed using Fourier-transform infrared (FTIR) spectroscopy, as illustrated in Figure 2. The infrared spectra of the samples obtained via the formic acid demineralization process show slight differences from those previously reported for the acetic acid process [21]. For the raw chitin (RCH) sample, the FTIR spectra display the three characteristic amide peaks of α-chitin [34]. Specifically, the characteristic peak amide I, associated with C=O stretching vibrations, was observed at 1627 cm^−1^. The characteristic peaks of amide II and amide III, corresponding to N-H bending and C-N stretching, were observed at 1509 cm^−1^ and 1392 cm^−1^, respectively [35]. A broad absorption band centered around 3440 cm^−1^ is assigned to the O-H stretching vibrations [36]. Notably, the N-H stretching region shows a distinct split into two peaks at 3274 cm^−1^ and 3068 cm^−1^, similar to the spectra of chitin reported in previous work by Focher et al. [37] Other vibrational peaks, including C-H stretching (2933 cm^−1^), H-C-H bending (1448 cm^−1^), and the asymmetric C-O-C glycosidic bridge stretching (1166 cm^−1^), further confirm the successful extraction of chitin from the mussel shell [38,39]. The spectra for purified chitin (PCH) remained largely consistent with the RCH profiles, with minor shifts in peak positions, confirming that the deproteinization and decolorization treatments successfully removed non-chitin components without degrading the biopolymer’s functional groups. The FT-IR bands observed in this study are in good agreement with those reported for chitin and chitosan derived from crustacean and insect sources [34,40].

Upon conversion of PCH to CTS via deacetylation, the FTIR spectra exhibit the characteristic functional groups of chitosan, with notable wavenumber shifts. The characteristic Amide I, II, and III bands in the CTS samples were observed at 1625 cm^−1^, 1511 cm^−1^, and 1369 cm^−1^, respectively [35]. The O-H stretching vibration was observed at 3457 cm^−1^, while the N-H stretching peaks were observed at 3274 cm^−1^ and 3056 cm^−1^ [35,41]. Consistent with the chitin structure, C-H stretching, H-C-H bending, asymmetric stretching of the C-O-C bridge, C-O stretching, and H-C-H out-of-plane bending are also observed at 2931, 1450, 1164, 1010, and 849 cm^−1^, respectively [35,41]. From these results, it can be noted that while the demineralization agent was changed to formic acid, the functional groups of the resulting biopolymers were preserved, yielding FTIR spectra of chitin and chitosan similar to those from crustacean and insect sources [34,40].

**Figure 3:**
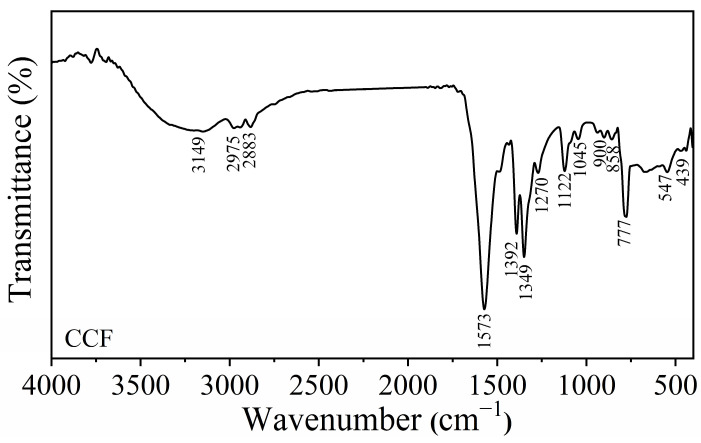
FTIR spectrum of calcium formate (CCF) derived from the demineralization effluent of mussel shells.

The FTIR spectrum of calcium formate (CCF), produced from the demineralization effluent of mussel shells using formic acid, is presented in Figure 3. The FTIR spectrum of CCF exhibits the characteristic vibrational modes of the formate group (HCOO^−^) and the metal-oxygen (Ca-O). The C-H stretching vibration, typical of the formate group, was identified as a weak peak at 2975 and 2883 cm^−1^. The sharp peaks at 1573 cm^−1^ and 1346 cm^−1^ are assigned to the asymmetric and symmetric C-O stretching modes, respectively. The vibrational peaks at 1392 and 777 cm^−1^ are assigned to the bending vibration of O−C−H and O=C−O, respectively [42]. Moreover, consistent with the report by Dechapinan et al. [43] for calcium salts derived from biogenic sources, a broad band was observed in the low-frequency range between 400–600 cm^−1^, which is assigned to the Ca-O stretching vibrations, confirming the formation between calcium (Ca^2+^) and formate (HCOO^−^) ions. In addition, a broad absorption peak observed at approximately 3149 cm^−1^ is attributed to the O-H stretching vibrations of residual moisture [44]. These results are in line with the FTIR spectra of calcium formate (Ca(HCOO)_2_) reported in the previous studies [45], confirming the conversion of mussel shell calcium carbonate to calcium formate.

### 2.4. Crystal Structure Analysis via XRD

**Figure 4:**
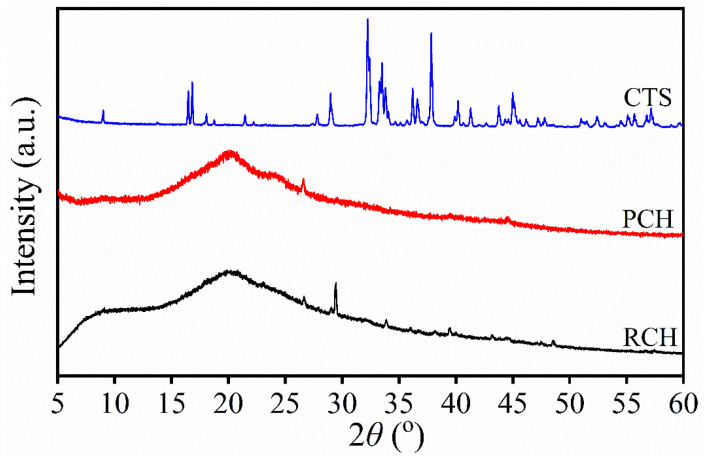
XRD patterns of raw chitin (RCH), purified chitin (PCH), and chitosan (CTS) samples extracted from mussel shells.

The XRD patterns of raw chitin (RCH), purified chitin (PCH), and chitosan (CTS) extracted from mussel shells were investigated by X-ray diffraction (XRD) and are shown in Figure 4. All XRD patterns were recorded over a 2θ range of 5–60°. The XRD patterns of RCH and PCH extracted using formic acid are similar to those extracted using acetic acid in our previous work [21], whereas the XRD pattern of chitosan is slightly different. The XRD patterns of both RCH and PCH show two broad diffraction reflections at 2θ 9.0° and 20.2°. These peaks correspond to the (020) and (110) crystallographic planes, respectively, consistent with the results reported in the XRD patterns of chitin extracted from crustaceans and insects [29,46]. However, after deacetylation in a strong alkaline solution, the XRD pattern of the resulting chitosan (CTS) showed very small diffraction peaks of chitosan, expected at approximately 2θ ≈ 10° and 20°, are present but exhibit very low intensity. This behavior can be attributed to the presence of relatively more crystalline phases of inorganic residues, which dominate the diffraction pattern due to their higher crystallinity. A similar presence of crystalline peaks was also observed in the previous study using the same preparation route [21]. However, in the present work, the relative intensity of these crystalline peaks is higher, resulting in a significant suppression of the characteristic amorphous diffraction features of chitosan.

**Figure 5:**
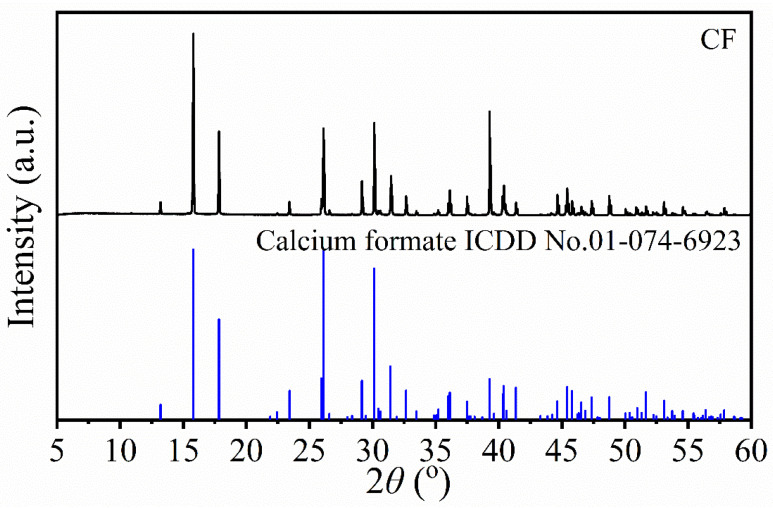
XRD pattern of calcium formate (CCF) derived from the demineralization effluent of mussel shells.

The crystalline structure of calcium formate (CCF) produced from mussel shell demineralization effluent using formic acid was investigated by X-ray diffraction (XRD), as shown in Figure 5. The resulting diffractogram was compared with the international diffraction databases to verify its chemical structure. The diffraction pattern obtained in this study was found to be in excellent agreement with the standard ICDD card no.01-074-6923, which corresponds to the orthorhombic crystal system of anhydrous calcium formate (Ca(HCOO)_2_). The diffraction pattern of calcium formate in this work corresponds to that reported previously by Hassan et al. [47], which produces calcium formate from CaCO_3_ in sludge from the paper production process. The results from both FTIR and XRD analysis of CCF confirm that the demineralization solution (DMS) obtained from the formic acid treatment of mussel shells was successfully transformed into anhydrous calcium formate (Ca(HCOO)_2_).

### 2.5. Thermal Behavior via TGA

**Figure 6:**
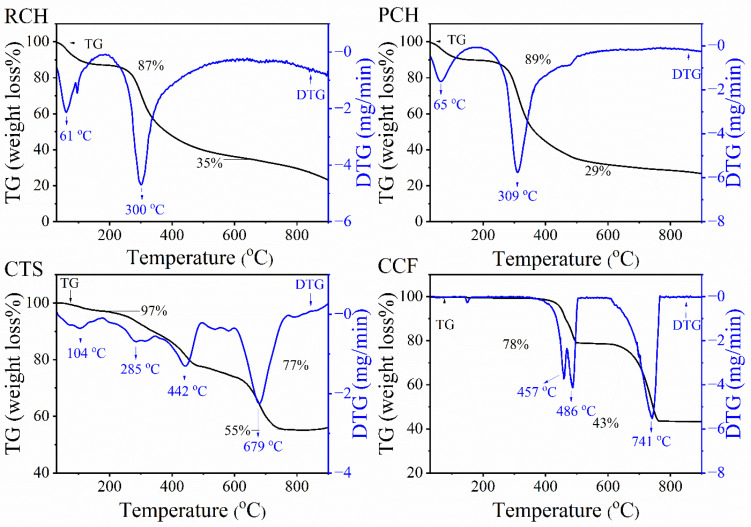
TG/DTG curves of raw chitin (RCH), purified chitin (PCH), chitosan (CTS), and calcium formate (CCF) samples extracted from mussel shells.

TG/DTG curves of raw chitin (RCH), purified chitin (PCH), and chitosan (CTS) extracted from mussel shells were analyzed using thermogravimetric analysis (TGA), as illustrated in Figure 6. The thermal decomposition behavior of RCH, PCH, and CTS derived from mussel shells using formic acid during the demineralization step was investigated by TG/DTG analysis over a temperature range of 30–900 °C. The obtained profiles differ from those previously reported for samples prepared with acetic acid during demineralization [21]. For RCH, the initial weight loss occurred within 30–185 °C, exhibiting a DTG peak at 61 °C with an approximate mass loss of 13%, attributed to the removal of physically adsorbed water (H_2_O) within the chitin structure [48]. Subsequently, the second decomposition step was observed between 185–649 °C, with a DTG peak at 300 °C and a mass loss of about 52%. This stage is associated with the breakdown of the chitin polymer, involving depolymerization of polysaccharide chains, thermal cracking, ring-opening reactions, and degradation of acetylated units [49]. For PCH, a similar two-step decomposition pattern was observed, with slight variations following purification by deproteinization and decolorization. The TG/DTG curve of PCH exhibits the thermal decomposition pattern in the ranges 30–178 °C (DTG peak at 65 °C) and 178–647 °C (DTG peak at 309 °C), with 11% and 60% mass loss, respectively. The slight shift in thermal stability after deproteinization and bleaching indicates the successful removal of protein and pigments. Comparatively, the degradation temperature of chitin from mussel shells in this study is relatively lower than values reported for chitin from other sources, such as crab shell (372 °C) [50], shrimp shell (350 °C) [51], and insect (390 °C) [52], highlighting the impact of biological origin on thermal characteristics of biopolymers.

The thermal behavior of CTS exhibits an initial weight loss of approximately 3%, occurring between 30–177 °C (DTG peak at 104 °C), attributed to the evaporation of physically adsorbed and bound water molecules trapped within the hydrophilic chitosan structure [48]. Following that, a second decomposition step was observed in the range of 177–496 °C (DTG peak at 285 and 442 °C) with a mass loss of approximately 20%. This stage represents the synergistic degradation of the polymer backbone, encompassing the deamination of amino groups, which releases volatile ammonia (NH_3_), alongside polysaccharide depolymerization, thermal cracking, and pyrolytic ring-opening [49,53]. Notably, the mass loss observed during this phase was lower than that of PCH, as the acetyl groups (COCH_3_) had been substantially eliminated during the deacetylation process. The third decomposition step was identified between 496 and 771 °C (DTG peaks at 679 °C), with 22% mass loss corresponding to the carbonization of residual organic matter and the emission of volatile fatty acids (C_2_-C_6_) [53,54]. The cumulative analysis revealed a final residual mass of approximately 55%, which is lower than values previously reported for shrimp-derived chitosan (30–33%) [53].

The thermal behavior of CCF produced from the demineralization effluent of mussel shells using formic acid was also illustrated in Figure 6. The TG/DTG curves reveal a distinct two-stage mass-loss step, corresponding to the thermal decomposition of calcium formate, as reported in previous studies [55]. The first thermal decomposition step was initiated in the temperature range between 342–505 °C (DTG peaks at 457 and 486 °C), corresponding to the transformation of anhydrous calcium formate (Ca(HCOO)_2_) into calcium carbonate (CaCO_3_) and the release of volatile formaldehyde (HCHO), as represented in Equation (2) [55,56]. In this step, the observed mass loss for the CCF samples was approximately 22%, aligning closely with the theoretical mass loss of 23.08%.

Ca(HCOO)_2_(s) → CaCO_3_(s) + HCHO(g) (2)

The second decomposition step occurred between 601–766 °C (DTG peaks at 741 °C), corresponding to the thermal decomposition of calcium carbonate (CaCO_3_), resulting in the formation of calcium oxide (CaO) and the elimination of carbon dioxide (CO_2_) according to Equation (3) [55,56]. The experimental mass loss recorded during this second stage was approximately 35%, close to the theoretical value of 33.85%.

CaCO_3_(s) → CaO(s) + CO_2_(g) (3)

Overall, the total mass remaining was about 43% (>766 °C), is highly consistent with that of calcium formate synthesized from cockle shells reported by Wang et al. (46%) [57] and calcium formate from paper processing sludge by Hassan et al. (42%) [47]. This high degree of correlation confirms the successful synthesis of anhydrous calcium formate (Ca(HCOO)_2_).

### 2.6. Morphologies via SEM

**Figure 7:**
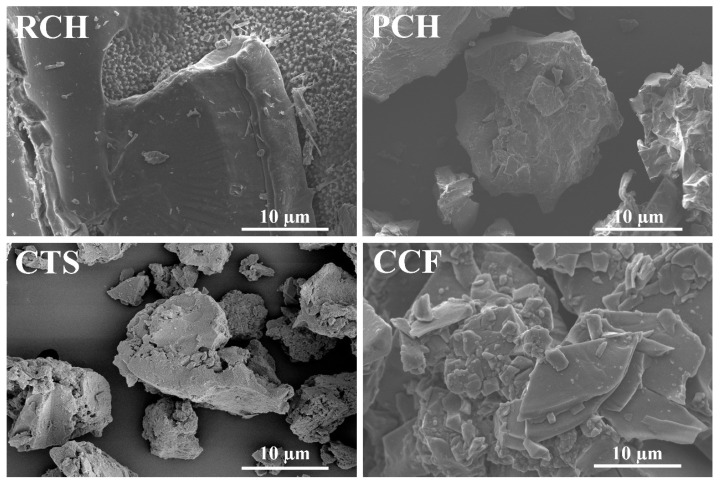
SEM images of raw chitin (RCH), purified chitin (PCH), chitosan (CTS), and calcium formate (CCF) samples extracted from mussel shells.

The surface morphologies and microstructural characteristics of the synthesized RCH, PCH, CTS, and CCF were examined using a scanning electron microscope (SEM), as illustrated in Figure 7. The SEM images of products obtained via the formic acid demineralization process exhibit different morphologies compared with those obtained via the acetic acid process [21]. The SEM image of raw chitin (RCH) reveals numerous microparticles of various dimensions on the surface. However, following sequential purification via deproteinization and decolorization, the purified chitin (PCH) exhibits a significantly smoother surface, indicating the effective removal of residual impurities. This transition to a well-defined microstructure with reduced surface roughness is consistent with the morphology of chitin extracted from crustacean sources, as reported by Mohan et al. [58]. For chitosan (CTS), the SEM micrograph reveals a relatively shapeless porous-like surface morphology. A similar trend in morphological transformation, from rough and heterogeneous surfaces in raw chitin (RCH), to smoother surfaces of purified chitin (PCH), and finally to more porous surfaces in CTS, has also been reported for chitosan derived from shrimp shells by de Queiroz et al. [59].

The morphology of the CCF produced from the demineralization effluent of mussel shells using formic acid reveals plate-like crystals of varying sizes. This morphology differs significantly from that reported in previous studies, where calcium formate typically exhibits bipyramidal or polyhedral crystal structures. For instance, bipyramidal polyhedral crystals of calcium formate have been identified in sediments from Alkali Lake, Oregon, USA, as reported by de Chukanov et al. [45], while similar crystal habits were also observed in calcium formate derived from paper processing sludge, as described by Wang et al. [57].

## 3. Materials and Methods

### 3.1. Preparation of Raw Material and Reagents

Waste mussel shells (*Perna viridis*) were obtained from the Ang Sila fishery market in Chonburi, Thailand. To eliminate external impurities and the organic matrix, the shells were thoroughly cleaned with water. The cleaned shells were subsequently pulverized using a mortar and pestle, passed through a 100-mesh sieve to ensure uniform particle size, and thermally dried at 100 °C for 2 h. The elemental composition of the resulting mussel shell powders (MSP) was analyzed via X-ray Fluorescence (XRF), confirming a calcium content of 96%.

All chemical reagents utilized in this study were of analytical grade and used without further purification. Formic acid (HCOOH, 85%, Loba Chemie, Mumbai, India) was diluted to 1 M formic acid solution with deionized (DI) water for the demineralization process. For protein removal and deacetylation, sodium hydroxide pellets (NaOH, 99%, Merck, Darmstadt, Germany) were dissolved with deionized (DI) water to prepare 1 M and 10 M concentrations, respectively. For the pigment removal process, hydrogen peroxide (H_2_O_2_, 30%, Merck) was diluted with deionized (DI) water to prepare 10% *w*/*v* solution. Ethanol (99%, Carlo Erba, Cornaredo, Italy) served as the precipitating agent for chitosan recovery.

### 3.2. Biopolymer Extraction and Fraction Separation

The demineralization step involved the acid digestion of MSP to separate the inorganic matrix from the organic chitin. According to the stoichiometric relationship in Equation (4), 100 g of MSP was reacted with 2000 mL of 1 M formic acid. The mixture was stirred at 600 rpm using a magnetic stirrer at ambient temperature. The cessation of CO_2_ effervescence signaled the completion of the reaction, resulting in a distinct biphasic system: a solid residue (chitin-rich) and a solution (mineral-rich).

MSP (CaCO_3_ + Chitin) (s) + 2HCOOH(aq) → Ca^2+^(aq) + 2HCOO^−^(aq) + H_2_O(l) + CO_2_(g)↑ + Chitin(s) (4)

Vacuum filtration was employed to isolate the phases. The solid fraction, labeled Raw Chitin (RCH), was rinsed with 10 mL of distilled water to remove residual formic acid. The filtrate, containing dissolved calcium and formate ions (Demineralization Solution, DMS), was reserved for the further production of calcium formate.

### 3.3. Purification of Chitin (Deproteinization and Decolorization)

To obtain purified chitin (PCH), the RCH underwent a series of chemical treatments. First, protein constituents were eliminated by treating the RCH with 1 M NaOH at a 1:10 (*w*/*v*) ratio under constant stirring (600 rpm) for 1 h. The resulting Deproteinized Chitin (DPC) was separated from the alkaline solution by vacuum filtration. Subsequently, the DPC was bleached to remove residual pigments using a 10% *w*/*v* H_2_O_2_ solution (1:10 *w*/*v* ratio). This reaction was maintained at 90 °C for 0.5 h with continuous stirring. The deep yellow suspended solid, identified as Purified Chitin (PCH), was filtered, washed extensively with 10 mL of deionized water (three replicates), separated by vacuum filtration, and dried at 60 °C for 2 h.

### 3.4. Synthesis of Chitosan via Deacetylation

The transformation of PCH into chitosan (CTS) was performed through thermochemical deacetylation. PCH was soaked in a 10 M NaOH solution (1:20 *w*/*v*) and heated to 100 °C for 4 h with vigorous stirring. After cooling to room temperature, 20 mL of 99% ethanol was added to the slurry, and the mixture was stirred for 30 min to facilitate solidification and stabilization of the chitosan polymer chains. The CTS precipitates were collected via filtration, purified with 10 mL of 99% ethanol washes (three replicates), and dried at 60 °C for 2 h.

### 3.5. Production of Calcium Formate Powders

The DMS obtained from the demineralization process was used to produce calcium formate (CCF). The solution was subjected to controlled thermal evaporation in an oven at 60 °C for 24 h. The resulting crystalline solid was ground and sieved (100 mesh) to produce a fine white powder. The theoretical yield was calculated from the initial calcium concentration (0.96 M) determined by XRF analysis, assuming calcium was the limiting reactant during formic acid digestion.

### 3.6. Analytical Characterization

The physicochemical attributes of the synthesized RCH, PCH, CTS, and CCF were comprehensively evaluated using several analytical techniques. To identify functional groups and chemical bonds, Fourier Transform Infrared (FTIR) spectroscopy was performed using a PerkinElmer Spectrum GX instrument (Waltham, MA, USA). The spectra were captured within the wavenumber range of 4000–400 cm^−1^ at a resolution of 4 cm^−1^ over 32 scans. Each specimen (30 mg) was pulverized and mixed with spectroscopic-grade potassium bromide (KBr) to form a translucent pellet before measurement. The phase identification and crystal structure were investigated using X-ray diffraction (XRD) with a Rigaku MiniFlex diffractometer (Tokyo, Japan) equipped with a Cu Kα radiation source (operating at 40 kV and 32 mA). The diffraction patterns were recorded from 5° to 60° (2θ) with a consistent scanning rate of 0.04°/s. Furthermore, the thermal endurance and degradation behavior of the samples were analyzed using a PerkinElmer Pyris Diamond thermogravimetric analyzer (TGA). Approximately 30 mg of each sample was placed in an alumina (α-Al_2_O_3_) crucible and heated from 30 to 900 °C at a linear rate of 10 °C/min under a continuous nitrogen (N_2_) flow. Finally, the surface morphology and microstructural characteristics were examined using a Scanning Electron Microscope (SEM, LEO 1530, Atlanta, GA, USA). To prevent charging effects and ensure high-resolution imaging, all samples were sputter-coated with a thin gold film to provide adequate electrical conductivity prior to observation.

## 4. Conclusions

This study demonstrates a sustainable biorefinery approach for the concurrent extraction of chitin, chitosan, and calcium formate from mussel shell waste using formic acid as an efficient demineralizing agent. The comprehensive characterization using FTIR, XRD, TGA, and SEM confirmed that the prepared biopolymers and calcium compound exhibit chemical and physical properties consistent with those of materials derived from various natural sources. The chemical and structural integrity of the isolated biopolymers was confirmed by FTIR and XRD analyses, which exhibited the characteristic functional groups and crystal structures of chitin and its deacetylated derivative, chitosan. The physicochemical characterization confirmed that formic acid is an effective demineralizing agent for extracting chitin and chitosan from mussel shells, yielding biopolymer products with yields comparable to those reported in previous research. However, integrating the demineralization effluent into the subsequent production of calcium formate significantly enhances the process’s overall potential and efficiency. Furthermore, the developed process represents a significant advancement in the circular economy by eliminating the generation of wastewater typically found in traditional chitosan production while simultaneously converting industrial fishery food waste into multiple high-value products. By optimizing resource use and providing a zero-waste pathway, this integrated formic acid approach offers an environmentally responsible solution for the sustainable production of biopolymers and calcium compounds from marine bio-resources.

## Data Availability

The original contributions presented in this study are included in the article. Further inquiries can be directed to the corresponding authors.
